# Mitigating effects of desert dust storms in asthmatic children: health visitors’ perceptions, Cyprus

**DOI:** 10.1093/eurpub/ckac131.151

**Published:** 2022-10-25

**Authors:** P Kinni, P Kouis, M Charalambous, MG Kakkoura, SA Elia, E Kampriani, S Achilleos, A Panayiotou, N Middleton, PK Yiallouros

**Affiliations:** 1 Respiratory Physiology Laboratory, Medical School, University of Cyprus, Nicosia, Cyprus; 2 Educational Sector, Nursing Services, Ministry of Health, Nicosia, Cyprus; 3 Department of Population Health, University of Oxford, Oxford, UK; 4 Department of Social and Political Sciences, University of Cyprus, Nicosia, Cyprus; 5 Cyprus International Institute for Environmental and Public Health, Cyprus University of Technology, Limassol, Cyprus; 6 Department of Nursing, Cyprus University of Technology, Limassol, Cyprus; 7 Department of Primary Care and Population Health, University of Nicosia Medical School, Nicosia, Cyprus

## Abstract

**Background:**

Countries of the Mediterranean basin such as Cyprus are experiencing frequent desert dust storm (DDS) events that adversely impact children with asthma. As school health visitors (SHVs) have important role in asthma management, we examined SHVs practices and perceptions on asthma management and their level of engagement in school-based interventions to mitigate DDS- health effects.

**Methods:**

A descriptive cross-sectional survey was conducted among SHVs across state schools in Cyprus via an anonymous questionnaire, which rated the importance of asthma management measures (10-point scale), current implementation of these practices (1=never - 5=always), and regulatory authorities’ preparedness to respond to DDS events (1-5 Likert scale).

**Results:**

Responses from 78 of the 93 SHVs (84%), with an average work experience of 13.5 years (SD 7.3), revealed discordance between high perception of the importance of asthma management measures and their actual implementation, with poor scoring especially in assessment of asthma control (M = 2.4, SD = 1.5), tracking school absenteeism (M = 2.1, SD = 1.0) and monitoring of asthma triggers (M = 1.9, SD = 1.4). Any DDS-related measures (e.g. air quality real-time monitoring, warnings, recommendations, awareness campaigns, etc) were implemented very infrequently. Ratings of authorities’ preparedness were moderate (<3.5), and only slightly higher in the health versus the educational or other government services. SHVs who recognized the severity of DDS and potential impact on asthmatic children were more likely to support school-based interventions for DDS events (OR = 7.3, 95% CI: 2.1-25.3).

**Conclusions:**

Asthma management practices in school settings in Cyprus are suboptimal and responses during DDS are fragmented. While SHV’s awareness and support for interventions is high, this is not reflected in current practices. A concerted effort is needed for adoption of policies and implementation of action plans for DDS within school settings.

**Key messages:**

• Suboptimal asthma management policies and practices were found to be implemented in schools in Cyprus, a country highly exposed to dust events.

• School health visitors recognize the health impact of desert dust on asthmatic children and support the plan for dust-mitigation programmes in schools, despite authorities’ low preparedness.

